# Estimating Risk of Cardiovascular Disease Among Long-Term Colorectal Cancer Survivors: A Nationwide Cohort Study

**DOI:** 10.3389/fcvm.2021.721107

**Published:** 2022-01-17

**Authors:** Seogsong Jeong, Gyeongsil Lee, Seulggie Choi, Kyae Hyung Kim, Jooyoung Chang, Sung Min Kim, Kyuwoong Kim, Joung Sik Son, Yoosun Cho, Sang Min Park

**Affiliations:** ^1^Department of Biomedical Sciences, Seoul National University Graduate School, Seoul, South Korea; ^2^Department of Family Medicine, Seoul National University Hospital, Seoul, South Korea; ^3^National Cancer Control Institute, National Cancer Center, Goyang-si, South Korea; ^4^Department of Family Medicine, Korea University Guro Hospital, Seoul, South Korea; ^5^Total Healthcare Center, Kangbuk Samsung Hospital, Sungkyunkwan University School of Medicine, Seoul, South Korea

**Keywords:** cancer survivors, cardiovascular disease, gastrointestinal cancer, coronary heart disease, stroke

## Abstract

**Background:**

Concerns about a growing number of colorectal cancer survivors have emerged regarding cardiovascular disease (CVD) risks. However, there is not yet a predictive tool that can estimate CVD risk and support the management of healthcare as well as disease prevention in terms of CVD risk among long-term colorectal cancer survivors.

**Aim:**

To develop predictive tools to estimate individualized overall and each subtype of CVD risk using a nationwide cohort in South Korea.

**Methods and Results:**

A total of 4,709 newly diagnosed patients with colorectal cancer who survived at least 5 years in the National Health Insurance System were analyzed. Cox proportional hazard regression was used for the identification of independent risk factors for the derivation of predictive nomograms, which were validated in an independent cohort (*n* = 3,957). Age, fasting serum glucose, γ-glutamyl transpeptidase, Charlson comorbidity index, household income, body mass index, history of chemotherapy, cigarette smoking, and alcohol consumption were identified as independent risk factors for either overall CVD or each subtype of CVD subtype. Based on the identified independent risk factors, six independent nomograms for each CVD category were developed. Validation by an independent cohort demonstrated a good calibration with a median C-index of 0.687. According to the nomogram-derived median score, relative risks of 2.643, 1.821, 4.656, 2.629, 4.248, and 5.994 were found for overall CVD, ischemic heart disease, myocardial infarction, total stroke, ischemic stroke, and hemorrhage stroke in the validation cohort.

**Conclusions:**

The predictive tools were developed with satisfactory accuracy. The derived nomograms may support the estimation of overall and individual CVD risk for long-term colorectal cancer survivors.

## Introduction

Colorectal cancer is the third most common type of cancer for both men and women with an age-standardized incidence of 23.2 per 100,000 person-years. Even though it has increased by 9.5% between 1990 and 2017, age-standardized mortality has decreased by 13.5% globally (1, 2). In recent years, significant concerns have arisen as there may be an increased risk of cardiovascular disease (CVD) after cancer diagnosis due to shared risk factors, cardiotoxicity derived from cancer treatment, and the mechanisms associated with cancer biology (3–5).

To date, a number of studies have identified predictors of CVD, such as metabolic risk and lifestyle behaviors (6, 7). In addition, several tools were developed for estimation of cardiovascular risks, including atherosclerotic CVD risk score and Framingham risk score (8–10). However, such studies were not quantifiable for heterogeneities between cancer survivors and others (11). In addition, a case-control study of the UK Clinical Practice Research Datalink reported that survivors of most site-specific cancers have increased risk of CVD compared to the general population, and called for future strategies to minimize cardiovascular risk, which is necessary by considering the growing cancer survivor population (12).

Colorectal cancer survivors were previously found to have poor blood pressure control and a higher likelihood of hypertension, which are CVD-related chronic conditions (13). Considering the absence of a tool for the prediction of CVD risks for long-term colorectal cancer survivors despite the unmet need of reducing CVD risks, we developed prediction models based on the identified independent risk factors for each CVD, including overall CVD, ischemic heart disease (IHD), myocardial infarction (MI), total stroke (TS), is-chemic stroke (IS), and hemorrhage stroke (HS), which were visualized as nomograms for general use and validated in an independent population dataset from the National Health Insurance System (NHIS).

## Materials and Methods

### Study Population

This study is a Korean nationwide retrospective observational study. The Korean NHIS is a mandatory health insurance covering healthcare services, including outpatient and inpatient visits, laboratory examination, treatment, and pharmaceutical prescriptions for all Korean citizens (14). Citizens aged 40 years or more are eligible for a biannual health examination that involves self-reported questionnaires on medical history, lifestyle factors, anthropometric measurement, and laboratory examinations, and these data are provided by the NHIS for research purposes on request (15).

**Figure 1:**
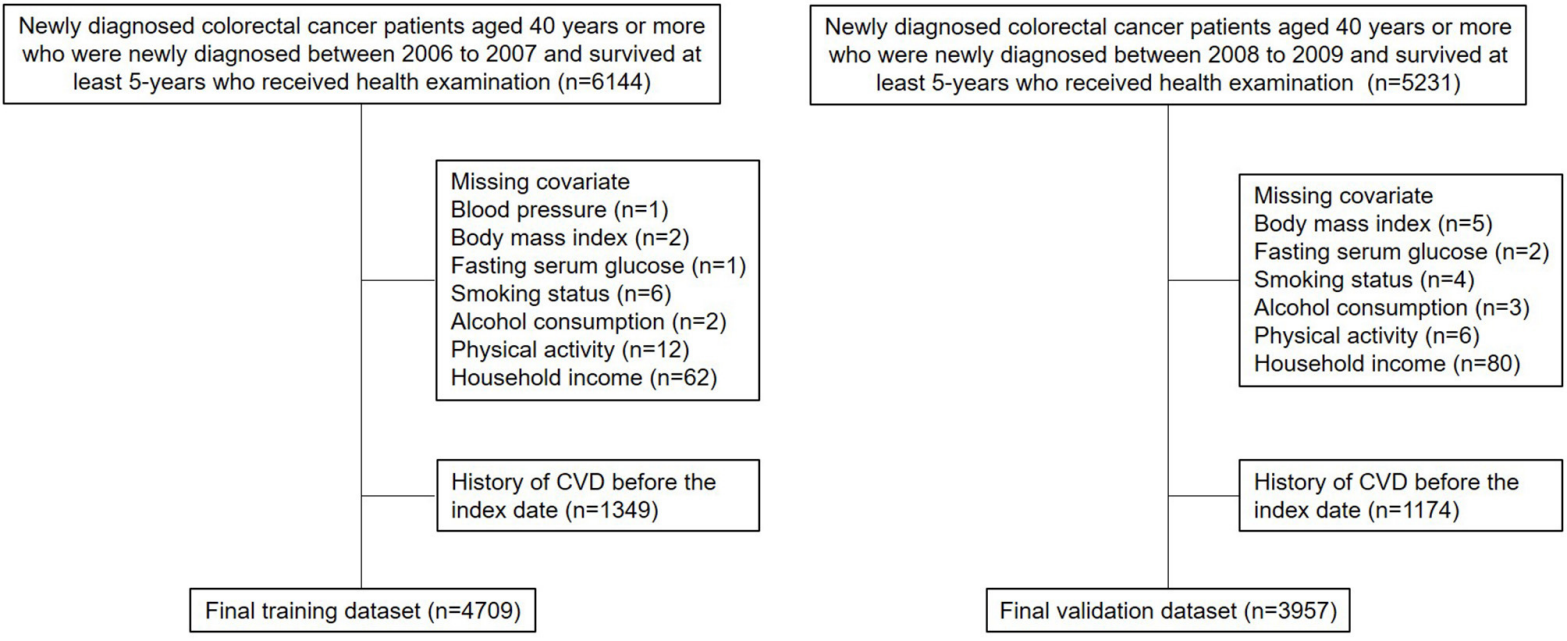
Participant inclusion flowchart.

There was a total of 6,144 (newly diagnosed with colorectal cancer between 2006 and 2007) and 5,231 (newly diagnosed with colorectal cancer between 2008 and 2009) survivors newly diagnosed with colorectal cancer who underwent national health screening within 2 years before the date of 5-year survival (Figure 1). Newly diagnosed colorectal cancer was identified using the ICD-10 code for colorectal cancer (C17-C21) and a critical condition code for cancer of the NHIS (16). The former group consisted of the training (2006-2007) cohort, whereas the latter group consisted of the validation (2008-2009) cohort. In addition, 1,349 and 1,174 patients were excluded due to CVD before the index date, and 86 and 100 patients were further excluded due to missing values, thus the results of this study may be biased to long-term colorectal cancer survivors who receive health examination. Finally, 4,709 and 3,957 patients consisted of the training and validation cohorts, respectively. A follow-up investigation was carried out until the date of CVD, death, or 31 December 2018. The graphical study design is shown in Supplementary Figure 1.

This study was approved by the Seoul National University Institutional Review Board (E-2004-191-1119) and conducted in accordance with the Transparent reporting of a multivariable prediction model for individual prognosis or diagnosis (TRIPOD) guidelines (17). The need for informed consent was waived as the NHIS database is anonymized under strict confidentiality guidelines. All authors had access to the study data and reviewed and approved the final manuscript.

### Primary Outcomes and Variables

The primary outcomes, including CVD (I20-I25, I60-I69), IHD (I20-I25), MI (I21-I24), TS (I60-I69), IS (I63), and HS (I60-I62), were defined as disease-specific hospitalization for ≥2 days with ICD-10 codes. The ICD-10 codes for CVDs were derived from the American Heart Association guidelines (18).

The following variables were evaluated in the univariate analysis for identification of independent predictors for CVDs: age (continuous, years), sex (categorical: men and women), systolic and diastolic blood pressure (continuous, mmHg), household income (categorical: 1st, 2nd, 3rd, and 4th quartiles), body mass index (BMI; continuous, kg/m2), fasting serum glucose (FSG) and total cholesterol (TC; continuous: mg/dL), liver function test, including aspartate aminotransferase (AST), alanine aminotransferase (ALT), and γ-glutamyl transpeptidase (γ-GT; continuous: IU/L), Charlson comorbidity index (CCI; continuous), smoking (categorical: never, previous, and current), alcohol consumption (categorical: never, 1-2, 3-4, and ≥5 day/week), physical activity, including walking and moderate-to-vigorous physical activity (MVPA; categorical: never, 1-2, 3-4, and ≥5 day/week), and history of chemotherapy and radiotherapy (categorical: yes and no). The following variables were additionally evaluated in the univariate analysis in a categorical form to ensure detection of non-linear associations: blood pressure, BMI, FSG, and TC. Assessment of outcomes and predictors for the outcomes were unblinded.

### Statistical Analysis

Patients with any missing data were not included. Continuous and categorical variables are presented with median [interquartile range (IQR)] and number (%), respectively. The univariate analysis was performed using the Cox proportional hazards regression, which was presented with a hazard ratio (HR) with a 95% confidence interval (CI). Significant predictors identified in the univariate analysis were enrolled for the multivariate analysis. A more significant form was adopted between continuous and categorical forms if both forms were found significant. Independently significant factors identified by the multivariate analysis composed predictive nomograms for each CVD. The nomogram score was defined as the total point derived from the nomogram. Performance of the nomograms was independently validated and evaluated using receiver operating curves with area under curve (AUC) values and calibration curves with concordance index from the Somer's rank correlation test. Relative risks were calculated by the absolute risk of the high-risk group/low-risk group for an intuitive measure of differences defined by the median nomogram score. A *P*-value of < 0.05 was considered statistically significant. All statistical analyses were carried out using the SAS Enterprise Guide 7.1 (SAS Institute, Cary, NC, USA) and the R project for statistical computing (version, 3.6.3; www.r-project.org).

## Results

### Patient Characteristics

The patient demographics are shown in Table 1. A total of 4,709 patients were analyzed as the training cohort. For the independent validation cohort, 3,957 patients were eligible for the present study. The median age of the patients was 60 years (IQR, 53-67). Male distribution was higher compared to female. For both training and validation cohorts, the median CCI was 3 (IQR, 1-5). A majority of the patients was never smoker (training: *n* = 3,016, 64.0%; validation: *n* = 2,433; 61.5%) and non-drinker (training: *n* = 3,349, 71.1%; validation: *n* = 2,742; 69.3%). In addition, more than a half of the patients carried out MVPA regularly (training: *n* = 2,420, 51.4%; validation: *n* = 2,158; 54.5%). According to the cancer site, the colon contributed the most, followed by the rectum, rectosigmoid junction, small intestine, and anus.

**Table 1:** Baseline characteristics of the colorectal cancer survivors.

**Characteristic**	**Training (*n* = 4,709)**	**Validation (*n* = 3,957)**
Age, years	60 (53-67)	60 (53-67)
**Sex**, ***n*** **(%)**		
Male	2,718 (57.7)	2,332 (58.9)
Female	1,991 (42.3)	1,625 (41.1)
**Income**, ***n*** **(%)**		
First quartile	198 (4.2)	208 (5.3)
Second quartile	407 (8.6)	421 (10.6)
Third quartile	1,105 (23.5)	979 (24.7)
Fourth quartile	2,999 (63.7)	2,349 (59.4)
Systolic blood pressure, mmHg	125 (116-135)	125 (116-134)
Diastolic blood pressure, mmHg	78 (70-81)	77 (70-81)
Body mass index, kg/m^2^	23.6 (21.5-25.5)	23.7 (21.6-25.7)
Fasting serum glucose, mg/dl	98 (89-108)	99 (90-111)
Total cholesterol, mg/dL	192 (169-218)	190 (168-216)
Aspartate aminotransferase, IU/L	24 (20-29)	24 (20-30)
Alanine aminotransferase, IU/L	20 (15-27)	20 (15-27)
γ*-*glutamyl transpeptidase, IU/L	22 (16-35)	23 (16-36)
Charlson comorbidity index	3 (1-5)	3 (1-5)
**Smoking status**, ***n*** **(%)**		
Never	3,016 (64.0)	2,433 (61.5)
Previous	1,168 (24.8)	1,085 (27.4)
Current	525 (11.1)	439 (11.1)
**Alcohol consumption**, ***n*** **(%)**		
0 day/week	3,349 (71.1)	2,742 (69.3)
1-2 day/week	797 (16.9)	736 (18.6)
3-4 day/week	326 (6.9)	274 (6.9)
≥5 day/week	237 (5.0)	205 (5.2)
**Walking**, ***n*** **(%)**		
0 day/week	1,400 (29.7)	1,092 (27.6)
1-2 day/week	761 (16.2)	683 (17.3)
3-4 day/week	935 (19.9)	849 (21.5)
≥5 day/week	1,613 (34.3)	1,333 (33.7)
**MVPA**, ***n*** **(%)**		
0 day/week	2,289 (48.6)	1,799 (45.5)
1-2 day/week	614 (13.0)	530 (13.4)
3-4 day/week	626 (13.3)	539 (13.6)
≥5 day/week	1,180 (25.1)	1,089 (27.5)
History of chemotherapy	1,062 (22.6)	1,100 (27.8)
History of radiotherapy	375 (8.0)	411 (10.4)
**Cancer site**, ***n*** **(%)**		
Small intestine	84 (1.8)	68 (1.7)
Colon	2,498 (53.0)	2,420 (61.2)
Rectosigmoid junction	559 (11.9)	208 (5.3)
Rectum	1,601 (34.0)	1,277 (32.3)
Anus and anal canal	33 (0.7)	34 (0.9)
≥2 sites	66 (1.4)	50 (1.3)

*Data are median (interquartile range) unless indicated otherwise. Data may not add up to 100% due to rounding. Acronyms: MVPA, moderate-to-vigorous physical activity*.

### Identification of the Independent Predictors

In the univariate analysis for overall CVD, age, sex, systolic blood pressure, categorical blood pressure, FSG, γ-GT, CCI, alcohol consumption, walking, MVPA, and history of chemotherapy were identified as significant factors (Supplementary Table 1). In the multivariate analysis, age, categorical FSG, γ-GT, and CCI were found significant. For IHD, age, in-come, systolic blood pressure, BMI, FSG, γ-GT, CCI, MVPA, and history of chemotherapy were found significant (Supplementary Table 2). Among them, age, income, BMI, γ-GT, CCI, and history of chemotherapy were identified as independent predictors. For MI, age, sex, income, systolic blood pressure, CCI, walking, and MVPA were significant (Supplementary Table 3). In the multivariable analysis, age, sex, and income were identified as independent predictors. As for TS, age, sex, systolic blood pressure, FSG, TC, γ-GT, CCI, alcohol consumption, walking, and MVPA were found significant, and age, FSG, and γ-GT were identified as independent predictors (Supplementary Table 4). In the univariate analysis for IS, age, systolic blood pressure, FSG, γ-GT, CCI, smoking, walking, and MVPA were found significant (Supplementary Table 5). In addition, age, FSG, γ-GT, and smoking were identified as independent predictors. Furthermore, age and alcohol consumption were significantly indicative of hemorrhage risk and both factors were independently associated (Supplementary Table 6).

### Model Derivation

**Figure 2:**
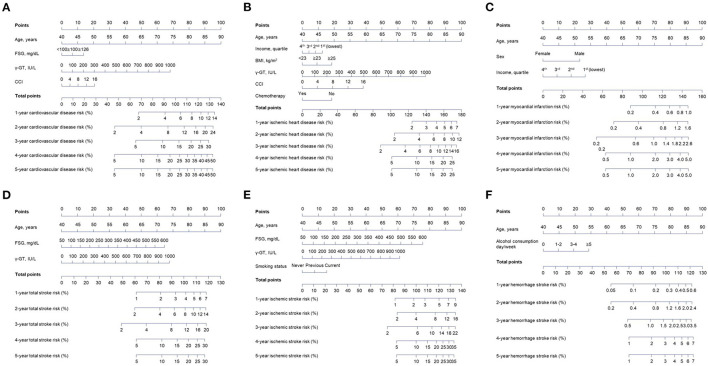
Derivation of the nomograms based on the identified independent risk factors for each cardiovascular disease subtype from the training cohort. An individualized evaluation was carried out by drawing straight vertical lines up to the assigned point for each risk factor. Corresponding risk probabilities are directly estimated by drawing a straight vertical line from the corresponding total point down to cardiovascular risk probabilities. **(A)** Overall cardiovascular disease nomogram. **(B)** Ischemic heart disease nomogram. **(C)** Myocardial infarction nomogram. **(D)** Total stroke nomogram. **(E)** Ischemic stroke nomogram. **(F)** Hemorrhage stroke nomogram.

As shown in Table 2, multivariate analyses were carried out to develop predictive nomograms. The four variables consisted of the nomogram for overall CVD (Figure 2). One-year and 5-year CVD risk could be over 14 and 50% for the highest risk, respectively. Six predictors formed the nomogram for IHD. According to the total points, IHD risk could exceed 25% for the highest total point individuals. A predictive nomogram for MI was developed with age, sex, and income. The five-year risk was over 5% for those with nomogram scores of at least 150. In the predictive nomogram for TS, all three variables, including age, FSG, and γ-GT highly contributed, and 5-year stroke risk could be over 30% for individuals with a nomogram score of at least 120. Four variables were included for the nomogram predicting IS. The contribution to the nomogram was highest for age, followed by FSG, γ-GT, and smoking. One-year and 5-year IS risks were designed to be over 9% and 35% for patients over 135 total points. As for the nomogram predictive of HS, 1-year and 5-year risks were over 0.6 and 7.0% for score ≥ 125, respectively.

**Table 2:** Multivariate analysis of significantly predictive factors for cardiovascular disease.

	**Input type**	**HR (95% CI)**	***P*-value**
**Cardiovascular disease**			
Age, years	Continuous	1.062 (1.051-1.073)	<0.001
Fasting serum glucose, mg/dl	Categorical	1.000 (Reference)	0.004
≥100 and <126	vs. <100	1.205 (0.991-1.465)	0.062
≥126	vs. <100	1.530 (1.180-1.983)	0.001
γ*-*glutamyl transpeptidase, IU/L	Continuous	1.002 (1.001-1.003)	<0.001
Charlson comorbidity index	Continuous	1.035 (1.003-1.067)	0.029
**Ischemic heart disease**			
Age, years	Continuous	1.052 (1.036-1.067)	<0.001
Income, quartile	Categorical	1.000 (Reference)	0.155
Second	vs. first	0.515 (0.263-1.010)	0.054
Third	vs. first	0.636 (0.369-1.095)	0.102
Fourth (highest)	vs. first	0.574 (0.348-0.949)	0.031
Body mass index	Categorical	1.000 (Reference)	0.010
≥23 kg/m^2^ and <25 kg/m^2^	vs. <23 *kg*/m^2^	1.346 (0.967-1.874)	0.078
≥25 kg/m^2^	vs. <23 *kg*/m^2^	1.589 (1.175-2.148)	0.003
γ*-*glutamyl transpeptidase, IU/L	Continuous	1.002 (1.000-1.004)	0.017
Charlson comorbidity index	Continuous	1.054 (1.007-1.102)	0.023
History of chemotherapy	vs. never	0.636 (0.443-0.912)	0.014
**Myocardial infarction**			
Age, years	Continuous	1.057 (1.024-1.092)	<0.001
Sex, female	vs male	0.527 (0.279-0.998)	0.049
Income, quartile	Categorical	1.000 (Reference)	0.154
Second	vs. first	0.312 (0.074-1.305)	0.111
Third	vs. first	0.515 (0.183-1.444)	0.207
Fourth (highest)	vs. first	0.356 (0.137-0.924)	0.034
**Total stroke**			
Age, years	Continuous	1.072 (1.058-1.086)	<0.001
Fasting serum glucose, mg/dl	Continuous	1.004 (1.001-1.007)	0.010
γ*-*glutamyl transpeptidase, IU/L	Continuous	1.002 (1.001-1.004)	<0.001
**Ischemic stroke**			
Age, years	Continuous	1.088 (1.068-1.109)	<0.001
Fasting serum glucose, mg/dl	Continuous	1.006 (1.002-1.009)	<0.001
γ*-*glutamyl transpeptidase, IU/L	Continuous	1.003 (1.001-1.004)	0.002
Smoking status	Categorical	1.000 (Reference)	0.016
Previous	vs. never	1.405 (0.973-2.028)	0.070
Current	vs. never	1.885 (1.184-2.998)	0.008
**Hemorrhage stroke**			
Age, years	Continuous	1.077 (1.037-1.119)	<0.001
Alcohol consumption	Categorical	1.000 (Reference)	0.002
1-2 day/week	vs. 0 day/week	0.841 (0.319-2.222)	0.727
3-4 day/week	vs. 0 day/week	5.525 (2.254-13.543)	<0.001
≥5 day/week	vs. 0 day/week	1.603 (0.379-6.788)	0.522

*HR calculated by Cox proportional hazard regression. HR, hazard ratio; CI, confidence interval*.

### Example

For example, the total score for an 80-year-old male with income of 1st quartile is 80 (for age)+23 (for male)+26 (for income) = 129 in the MI-predictive nomogram. Therefore, this patient has approximately 0.6 and 3.0% of 1-year and 5-year MI risks, respectively. If FSG, γ-GT, and CCI of this patient are <100 mg/dl, 500 IU/L, and 4, then the total point for the CVD nomogram is 80 (for age)+0 (for FSG)+34 (for γ-GT)+5 (for CCI) = 119, which indicates around 9, 24, and 37% of cardiovascular risk at 1-year, 3-year, and 5-year after the index date.

### Model Validation

**Figure 3:**
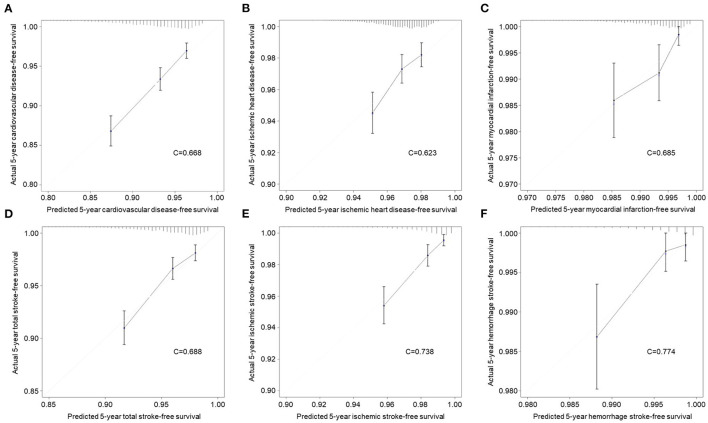
Calibration curves for evaluation of consistency between actual and predicted risks of cardiovascular diseases in the validation cohort. **(A)** Overall cardiovascular disease. **(B)** Ischemic heart disease. **(C)** Myocardial infarction. **(D)** Total stroke. **(E)** Ischemic stroke. **(F)** Hemorrhage stroke.

The validation of the sensitivity and specificity on the performance of the nomograms using receiver operating curve with AUC in the independent validation cohort is shown in Supplementary Figure 2. The performance of the nomograms was measured well by the AUC in the validation cohort (0.671 for CVD nomogram; 0.623 for IHD; 0.684 for MI; 0.690 for TS; 0.744 for IS; 0.774 for HS). The nomograms were also well-calibrated with calibration slopes of nearly 1 and C-indices of 0.668 for CVD, 0.623 for IHD, 0.685 for MI, 0.688 for TS, 0.738 for IS, and 0.774 for HS (Figure 3). For a description of the differences between high and low scores derived from the nomograms, all patients in the validation cohort were stratified according to the median nomogram score. Kaplan-Meier curves for risk of CVDs are shown in Supplementary Figure 3. In the validation cohort, the high-risk group revealed 10.8% of CVD (*n* = 214), whereas it was 4.1% (*n* = 81) for the low-risk group, which is 2.643 of relative risk (Table 3). In addition, the between-group relative risk of the high-risk groups was 1.821, 4.656, 2.629, 4.248, and 5.994 for IHD, MI, TS, IS, and HS, respectively.

**Table 3:** Median nomogram point-stratified cardiovascular disease event distribution in validation cohort.

	***N***	**Nomogram total point, median (IQR)**	**Event (%)**	**Relative risk**
**Cardiovascular disease**				
High risk	1,978	68.043 (60.253-77.339)	214 (10.8)	2.643
Low risk	1,979	37.042 (27.312-45.435)	81 (4.1)	Reference
**Ischemic heart disease**				
High risk	1,979	87.220 (78.801-98.069)	82 (4.1)	1.821
Low risk	1,978	53.121 (40.569-62.582)	45 (2.3)	Reference
**Myocardial infarction**				
High risk	2,049	76.633 (67.010-86.633)	25 (1.2)	4.656
Low risk	1,908	41.623 (30.811-50.811)	5 (0.3)	Reference
**Total stroke**				
High risk	1,978	63.878 (56.313-72.310)	134 (6.8)	2.629
Low risk	1,979	34.607 (25.498-42.028)	51 (2.6)	Reference
**Ischemic stroke**				
High risk	1,979	68.764 (61.101-77.326)	68 (3.4)	4.248
Low risk	1,978	39.409 (29.416-47.243)	16 (0.8)	Reference
**Hemorrhage stroke**				
High risk	1,923	58.000 (52.000-68.000)	17 (0.9)	5.994
Low risk	2,034	30.000 (21.468-38.000)	3 (0.1)	Reference

*IQR, interquartile range*.

## Discussion

Given the growing population and high incidence of CVD among colorectal cancer survivors, the availability of prediction scoring would be of interests to clinical decision support. However, limited information was available for assessing CVD risk for long-term colorectal cancer survivors. In the present study, we developed nomograms, an easily adopted tool that directly allows the prediction of disease risk by drawing lines down disease-free survival for each individual based on the independent predictors identified. In addition, validation by an independent cohort revealed the derived nomograms to be well-calibrated between predicted risk and actual risk.

To the best of our knowledge, this study is the first attempt to develop long-term colorectal cancer survivor-specific models for the prediction of CVD risk. The clinical importance of minimizing CVD risk among long-term colorectal cancer survivors may be facilitated through the use of prediction models, which estimate the risk of CVDs based on health examination records. In South Korea, all citizens aged 40 or more, including long-term colorectal cancer survivors, are mandatory to undergo national health screening biannually, and the development of a CVD prediction model applies to long-term colorectal cancer survivors without data unavailability. Meanwhile, CVD prediction models, such as the Framingham CVD risk score, have been regularly used in real-world management for the prevention of CVD (9). However, considering different baseline CVD risk and individualized characteristics, long-term colorectal cancer survivors were alienated from the prediction of CVD (12). Therefore, this study would light on the precision prediction of CVDs specifically for long-term colorectal cancer survivors. In addition, considering the clinical importance of the assessment of the predictive ability of prediction models, future studies conducted based on external cohorts may promote greater management of CVD prediction for long-term colorectal cancer survivors (19).

In recent years, colorectal cancer has replaced infection-associated cancers as the second most common cancer in a number of countries, and the trends in incidence and survival of colorectal cancer have been suggested to be monitored (20). In the United States, the 5-year survival for pooled all-stage colorectal cancer has increased from approximately 50% to over 65% since the mid-1970s (21). In addition, Colorectal cancer survivors were found to have a higher likelihood of hypertension and worse blood pressure control, which may be associated with increased CVD risk compared to the general population (13). Considering the increasing burden of colorectal cancer survivors and high CVD risks, targeting colorectal cancer survivors against CVD risks has now become an important concern, which needs different approaches from the general population, and comprehensive identification of risk factors for the overall and each type of CVD may allow estimation of potential CVD risks along with the provision of supportive information in individualized preventive management.

To date, some publications have explored colorectal cancer patient-specific risk factors for incident CVD. A previous retrospective analysis of the Surveillance Epidemiology and End Results database has confirmed that older age, male sex, and elevated carcinoembryonic antigen level are independent risk factors of CVD-related mortality in patients with colorectal cancer (22). However, there remained comprehensive identification of CVD type-dependent risk factors, which were visualized as nomograms for practical uses, as well as the development of a predictive tool for precision medicine against CVD risks. Furthermore, nomograms, which are easy-to-use tools for clinicians and researchers for the prediction of disease incidence and prognosis, are currently widely applied for the prediction of survival in patients with malignancy (23). However, no such tool was previously developed to estimate CVD risks in long-term colorectal cancer survivors. Taken together, this study not only identified comprehensive CVD type-specific risk factors but also provides tools that allow the implementation of individualized CVD-risk estimation to support the management of preventive strategies and follow-up plans in clinical practice.

In the present study, the history of chemotherapy was found to be preventive for IHD. Traditionally, concerns existed that chemotherapies may lead to increased cardiovascular risks due to cardiotoxic treatment effects (24). A pooled analysis of five randomized studies showed that some chemotherapy regimens, including bevacizumab and panitumumab, are associated with a higher risk of cardiotoxicity compared to other 5-fluorouracil-based regimens (25). From our point of view, we excluded the participants with CVD events within 5 years since diagnosis of colorectal cancer to limit CVD events to incident CVD among 5-year colorectal cancer survivors, thus those who had CVD due to chemotherapy-associated cardiotoxicity were not included in the study population. Therefore, there may be a selection bias that the study population may be less susceptible to cardiotoxicity because only patients without CVD for 5 years were enrolled in the study population. Another potential contributor for the positive impact of chemotherapy against CVD is that amelioration of colorectal cancer status derived by chemotherapy may represent overall health status and subsequently contribute to a lower risk of CVD. Therefore, the CVD risk of this specific study population may not have been increased due to chemotherapy but lowered the risk, which awaits future studies to confirm. Interpretation of our data requires caution as the results do not support chemotherapy as a favorable factor against CVD in all colorectal cancer survivors. Lowered incident and CVD risk by chemotherapy are limited to our study population who had not developed CVD after chemotherapy within 5 years from the diagnosis of colorectal cancer, which awaits future studies to further confirm.

Another significant concern exists regarding the applicability of the preexisting CVD risk estimation tools developed in other populations. A recently developed risk prediction model from Japan for CVD based on the Suita study of 3,080 male and 3,470 female participants included age, sex, blood pressure, lipoprotein cholesterols, diabetes mellitus, smoking, and urinary protein as risk factors (26). Another study that pooled multiple cohorts developed a risk prediction equation for CVD by applying smoking, blood pressure, diabetes, and TC (27). However, all models were not developed for long-term colorectal cancer survivors (28). In addition, most of the previous models targeted overall CVD as the only outcome (29). Compared to the predictors identified in our study, although partial predictors overlapped with previous models developed in other populations, there were still substantial differences and our models further involved FSG, γ-GT, CCI, and household income. These differences seem to be associated with a long-term colorectal cancer survivor setting. It is also important to note that lifestyle factors, including smoking, alcohol consumption, and physical activity, which were found to be significantly associated with CVD risk, were not involved in the nomogram for prediction of overall CVD (30). In the present study, smoking was not found to a significant indicator of CVD despite inclusion as a potential variable in the univariate and multivariate analysis. Colorectal cancer survivors tend to follow healthcare and smoke and drink less, but the need for more exercise may have resulted in the differences in colorectal cancer survivors compared to the general population. In addition, the low proportion of current smokers may have contributed to the non-significant association, thus smoking should not be considered as a non-harmful factor.

There remain some underlying limitations. Firstly, we used data from the NHIS that has no cancer-related information, such as tumor stage, thus the involvement of cancer-related information has the potential to improve the predictive performance of the nomograms. Considering the limited cancer-related information, the study population was strictly limited to those with at least 5 years of survival after a colorectal cancer diagnosis, of which the colorectal cancer patients are less affected by cancer itself but still affected by a history of colorectal cancer (31). Secondly, we developed models in colorectal cancer patients who survived at least 5 years. The derived models may not apply to colorectal cancer patients who survived <5 years, which await future studies to confirm. Thirdly, all patients were from South Korea and our model requires external validation from other regions. In addition, the derived nomograms were not compared with preexisting models built in other populations considering variations in involvement and weights of predictors. Another limitation is that we divided training and validation cohorts according to the year of a colorectal cancer diagnosis. Training and validation of the nomograms in a contemporaneous cohort may lead to more robust results, which await future studies to confirm. Lastly, this study involved data collected from self-reported questionnaires, such as lifestyle behaviors, thus such data used in the nomogram are subject to recall bias.

In conclusion, we have developed predictive nomograms for overall and each CVD based on a large-scale health examination cohort using independent risk predictors. The derived prediction models were validated in an independent cohort and revealed to be well-calibrated between predicted and actual risks of CVD. This is the first long-term colorectal cancer survivor-specific prediction model for CVDs, which may support the estimation of CVD risk and implementation of individualized preventive management.

## Data Availability Statement

The datasets presented in this article are not readily available because data used in the present study were achieved from the NHIS. Researchers can apply for the data from the NHIS on research purposes. Requests to access the datasets should be directed to The Korean NHIS (https://nhiss.nhis.or.kr).

## Ethics Statement

The studies involving human participants were reviewed and approved by Seoul National University Institutional Review Board (E-2004-191-1119). Written informed consent for participation was not required for this study because the NHIS database is provided in an anonymized from according to the strict confidentiality guidelines.

## Author Contributions

SJ and SP: conceptualization. SJ, SC, JC, KyuK, and SK: methodology, software, and data curation. SJ: formal analysis. SJ, GL, SC, KyaK, JC, KyuK, JS, SK, YC, and SP: writing—original draft preparation and writing—review and editing. SP: supervision. All authors have read and agreed to the published version of the manuscript.

## Conflict of Interest

The authors declare that the research was conducted in the absence of any commercial or financial relationships that could be construed as a potential conflict of interest.

## Publisher's Note

All claims expressed in this article are solely those of the authors and do not necessarily represent those of their affiliated organizations, or those of the publisher, the editors and the reviewers. Any product that may be evaluated in this article, or claim that may be made by its manufacturer, is not guaranteed or endorsed by the publisher.
