# Supplementary material for: Estimating Risk of Cardiovascular Disease Among Long-Term Colorectal Cancer Survivors: A Nationwide Cohort Study
Source: Front Cardiovasc Med. 2022 Jan 17;8:721107. doi: 10.3389/fcvm.2021.721107 (PMC8801493; doi:10.3389/fcvm.2021.721107)
Supplement: Supplementary file 1 [file Data_Sheet_1.PDF]

Supplementary Material

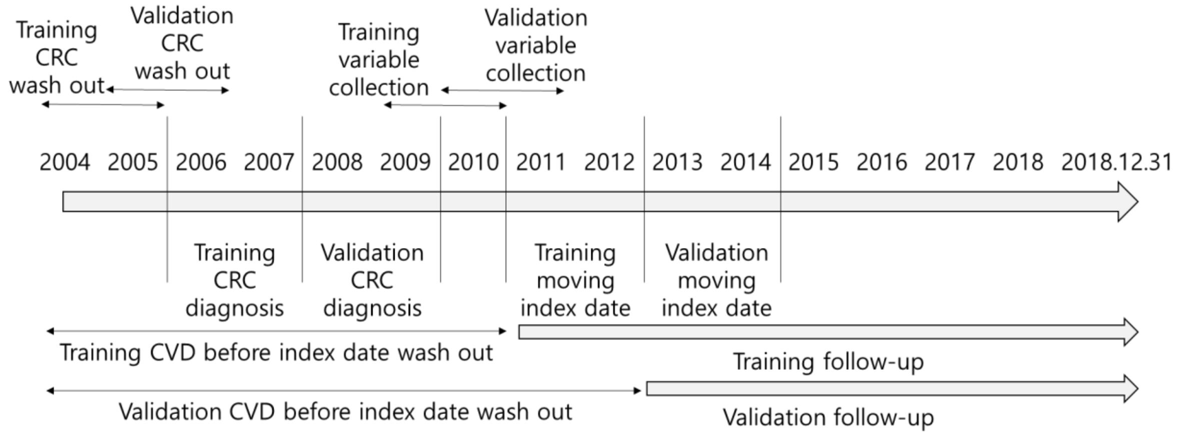

Supplementary Figure 1. Overall study design and periods of study cohorts, variable collection, and follow-up investigation.

Supplementary Table 1. Univariate and multivariate analysis of factors associated with cardiovascular disease

Variable	Input type	Univariate	Multivariate	
		P value	HR (95% CI)	P value
Age, years	Continuous	<0.001	1.057 (1.046-1.068)	<0.001
Sex, female	vs male	0.006	0.884 (0.720-1.087)	0.243
Income, quartile	Categorical	0.459	Not significant	
Second	vs first	0.818	Not significant	
Third	vs first	0.407	Not significant	
Fourth	vs first	0.244	Not significant	
SBP, mmHg	Continuous	<0.001	1.005 (1.000-1.011)	0.061
DBP, mmHg	Continuous	0.337	Not significant	
Blood pressure, mmHg	Categorical	0.002	Substituted by continuous	
SBP 120-129 and DBP<80	vs <120 and <80	0.019	Substituted by continuous	
SBP 130-139 or DBP 80-89	vs <120 and <80	0.001	Substituted by continuous	
SBP ≥140 or DBP ≥90	vs <120 and <80	<0.001	Substituted by continuous	
Body mass index, kg/m^2	Continuous	0.288	Not significant	
Body mass index	Categorical	0.203	Not significant	
≥23 kg/m^2 and <25 kg/m^2	vs <23 kg/m^2	0.446	Not significant	
≥25 kg/m^2	vs <23 kg/m^2	0.074	Not significant	
Fasting serum glucose, mg/dL	Continuous	0.001	Substituted by categorical	
Fasting serum glucose, mg/dL	Categorical	<0.001	1.000 (Reference)	0.013
≥100 and <126	vs <100	0.005	1.170 (0.961-1.424)	0.117
≥126	vs <100	<0.001	1.469 (1.130-1.909)	0.004
Total cholesterol, mg/dL	Continuous	0.050	Not significant	
Total cholesterol, mg/dL	Categorical	0.137	Not significant	
≥200 and <240	vs <200	0.053	Not significant	
≥240	vs <200	0.373	Not significant	
AST, IU/L	Continuous	0.591	Not significant	
ALT, IU/L	Continuous	0.599	Not significant	
γ-GT, IU/L	Continuous	<0.001	1.002 (1.000-1.003)	0.008
Charlson comorbidity index	Continuous	<0.001	1.042 (1.009-1.075)	0.012
Smoking status	Categorical	0.183	Not significant	
Previous	vs never	0.191	Not significant	
Current	vs never	0.117	Not significant	
Alcohol consumption	Categorical	0.031	1.000 (Reference)	0.729
1-2 day/week	vs 0 day/week	0.725	1.106 (0.868-1.408)	0.415
3-4 day/week	vs 0 day/week	0.108	1.201 (0.771-1.873)	0.418
≥5 day/week	vs 0 day/week	0.014	1.160 (0.755-1.784)	0.498
Walking	Categorical	0.010	1.000 (Reference)	0.811
1-2 day/week	vs 0 day/week	0.003	0.891 (0.692-1.147)	0.371
3-4 day/week	vs 0 day/week	0.113	0.980 (0.736-1.304)	0.889
≥5 day/week	vs 0 day/week	0.902	0.994 (0.780-1.267)	0.961
MVPA	Categorical	<0.001	1.000 (Reference)	0.294
1-2 day/week	vs 0 day/week	<0.001	0.844 (0.658-1.083)	0.183
3-4 day/week	vs 0 day/week	0.006	0.788 (0.601-1.034)	0.085
≥5 day/week	vs 0 day/week	0.296	0.925 (0.641-1.334)	0.676
History of chemotherapy	vs never	0.017	0.808 (0.639-1.022)	0.076
History of radiotherapy	vs never	0.363	Not significant	

HR calculated by Cox proportional hazard regression. Both continuous and categorical levels were analyzed in partial variables to identify significance of linear and non-linear associations. Acronyms: HR, hazard ratio; CI,

confidence interval; SBP, systolic blood pressure; DBP, diastolic blood pressure; AST, aspartate aminotransferase; ALT, alanine aminotransferase; γ-GT, γ-glutamyl transpeptidase; MVPA, moderate-to-vigorous physical activity.

Supplementary Table 2. Univariate and multivariate analysis of factors associated with ischemic heart disease

Variable	Input type	Univariate	Multivariate	
		P value	HR (95% CI)	P value
Age, years	Continuous	<0.001	1.048 (1.032-1.064)	<0.001
Sex, female	vs male	0.080	Not significant	
Income, quartile	Categorical	0.112	1.000 (Reference)	0.144
Second	vs first	0.030	0.503 (0.256-0.986)	0.045
Third	vs first	0.051	0.621 (0.360-1.070)	0.086
Fourth	vs first	0.022	0.569 (0.344-0.942)	0.028
SBP, mmHg	Continuous	<0.001	1.004 (0.996-1.013)	0.328
DBP, mmHg	Continuous	0.499	Not significant	
Blood pressure, mmHg	Categorical	0.011	Substituted by continuous	
SBP 120-129 and DBP<80	vs <120 and <80	0.003	Substituted by continuous	
SBP 130-139 or DBP 80-89	vs <120 and <80	0.005	Substituted by continuous	
SBP ≥140 or DBP ≥90	vs <120 and <80	0.015	Substituted by continuous	
Body mass index, kg/m^2	Continuous	0.029	Substituted by categorical	
Body mass index	Categorical	0.015	1.000 (Reference)	0.032
≥23 kg/m^2 and <25 kg/m^2	vs <23 kg/m^2	0.112	1.325 (0.950-1.848)	0.098
≥25 kg/m^2	vs <23 kg/m^2	0.004	1.501 (1.103-2.043)	0.010
Fasting serum glucose, mg/dL	Continuous	0.223	Not significant	
Fasting serum glucose, mg/dL	Categorical	0.007	1.000 (Reference)	0.013
≥100 and <126	vs <100	0.023	1.208 (0.911-1.603)	0.189
≥126	vs <100	0.005	1.348 (0.916-1.985)	0.130
Total cholesterol, mg/dL	Continuous	0.320	Not significant	
Total cholesterol, mg/dL	Categorical	0.413	Not significant	
≥200 and <240	vs <200	0.200	Not significant	
≥240	vs <200	0.953	Not significant	
AST, IU/L	Continuous	0.311	Not significant	
ALT, IU/L	Continuous	0.339	Not significant	
γ-GT, IU/L	Continuous	0.032	1.002 (1.000-1.003)	0.043
Charlson comorbidity index	Continuous	0.001	1.048 (1.001-1.097)	0.044
Smoking status	Categorical	0.129	Not significant	
Previous	vs never	0.099	Not significant	
Current	vs never	0.122	Not significant	
Alcohol consumption	Categorical	0.779	Not significant	
1-2 day/week	vs 0 day/week	0.613	Not significant	
3-4 day/week	vs 0 day/week	0.996	Not significant	
≥5 day/week	vs 0 day/week	0.335	Not significant	
Walking	Categorical	0.216	Not significant	
1-2 day/week	vs 0 day/week	0.051	Not significant	
3-4 day/week	vs 0 day/week	0.188	Not significant	
≥5 day/week	vs 0 day/week	0.563	Not significant	
MVPA	Categorical	0.030	1.000 (Reference)	0.378
1-2 day/week	vs 0 day/week	0.014	0.853 (0.611-1.191)	0.351
3-4 day/week	vs 0 day/week	0.029	0.738 (0.500-1.088)	0.125
≥5 day/week	vs 0 day/week	0.685	1.069 (0.659-1.734)	0.787
History of chemotherapy	vs never	0.010	0.644 (0.449-0.925)	0.017
History of radiotherapy	vs never	0.874	Not significant	

HR calculated by Cox proportional hazard regression. Both continuous and categorical levels were analyzed in partial variables to identify significance of linear and non-linear associations. Acronyms: HR, hazard ratio; CI,

confidence interval; SBP, systolic blood pressure; DBP, diastolic blood pressure; AST, aspartate aminotransferase; ALT, alanine aminotransferase; γ-GT, γ-glutamyl transpeptidase; MVPA, moderate-to-vigorous physical activity.

Supplementary Table 3. Univariate and multivariate analysis of factors associated with myocardial infarction

Variable	Input type	Univariate	Multivariate	
		P value	HR (95% CI)	P value
Age, years	Continuous	<0.001	1.045 (1.011-1.081)	0.010
Sex, female	vs male	0.033	0.469 (0.246-0.897)	0.022
Income, quartile	Categorical	0.163	1.000 (Reference)	0.182
Second	vs first	0.090	0.316 (0.075-1.328)	0.116
Third	vs first	0.147	0.499 (0.177-1.403)	0.187
Fourth	vs first	0.033	0.363 (0.139-0.945)	0.038
SBP, mmHg	Continuous	0.042	1.010 (0.992-1.028)	0.278
DBP, mmHg	Continuous	0.765	Not significant	
Blood pressure, mmHg	Categorical	0.391	Not significant	
SBP 120-129 and DBP<80	vs <120 and <80	0.501	Not significant	
SBP 130-139 or DBP 80-89	vs <120 and <80	0.313	Not significant	
SBP ≥140 or DBP ≥90	vs <120 and <80	0.620	Not significant	
Body mass index, kg/m^2	Continuous	0.447	Not significant	
Body mass index	Categorical	0.313	Not significant	
≥23 kg/m^2 and <25 kg/m^2	vs <23 kg/m^2	0.901	Not significant	
≥25 kg/m^2	vs <23 kg/m^2	0.160	Not significant	
Fasting serum glucose, mg/dL	Continuous	0.708	Not significant	
Fasting serum glucose, mg/dL	Categorical	0.861	Not significant	
≥100 and <126	vs <100	0.745	Not significant	
≥126	vs <100	0.728	Not significant	
Total cholesterol, mg/dL	Continuous	0.734	Not significant	
Total cholesterol, mg/dL	Categorical	0.740	Not significant	
≥200 and <240	vs <200	0.865	Not significant	
≥240	vs <200	0.493	Not significant	
AST, IU/L	Continuous	0.477	Not significant	
ALT, IU/L	Continuous	0.621	Not significant	
γ-GT, IU/L	Continuous	0.445	Not significant	
Charlson comorbidity index	Continuous	0.048	1.073 (0.973-1.183)	0.158
Smoking status	Categorical	0.387	Not significant	
Previous	vs never	0.490	Not significant	
Current	vs never	0.182	Not significant	
Alcohol consumption	Categorical	0.605	Not significant	
1-2 day/week	vs 0 day/week	0.524	Not significant	
3-4 day/week	vs 0 day/week	0.711	Not significant	
≥5 day/week	vs 0 day/week	0.207	Not significant	
Walking	Categorical	0.119	1.000 (Reference)	0.410
1-2 day/week	vs 0 day/week	0.043	0.630 (0.283-1.401)	0.257
3-4 day/week	vs 0 day/week	0.109	0.572 (0.221-1.481)	0.250
≥5 day/week	vs 0 day/week	0.135	0.557 (0.250-1.238)	0.151
MVPA	Categorical	0.168	1.000 (Reference)	0.707
1-2 day/week	vs 0 day/week	0.037	0.597 (0.247-1.439)	0.250
3-4 day/week	vs 0 day/week	0.272	0.785 (0.332-1.860)	0.583
≥5 day/week	vs 0 day/week	0.443	0.891 (0.249-3.187)	0.859
History of chemotherapy	vs never	0.374	Not significant	
History of radiotherapy	vs never	0.679	Not significant	

HR calculated by Cox proportional hazard regression. Both continuous and categorical levels were analyzed in partial variables to identify significance of linear and non-linear associations. Acronyms: HR, hazard ratio; CI,

confidence interval; SBP, systolic blood pressure; DBP, diastolic blood pressure; AST, aspartate aminotransferase; ALT, alanine aminotransferase; γ-GT, γ-glutamyl transpeptidase; MVPA, moderate-to-vigorous physical activity.

Supplementary Table 4. Univariate and multivariate analysis of factors associated with total stroke

Variable	Input type	Univariate	Multivariate	
		P value	HR (95% CI)	P value
Age, years	Continuous	<0.001	1.065 (1.050-1.080)	<0.001
Sex, female	vs male	0.034	0.916 (0.698-1.202)	0.527
Income, quartile	Categorical	0.346	Not significant	
Second	vs first	0.269	Not significant	
Third	vs first	0.522	Not significant	
Fourth	vs first	0.812	Not significant	
SBP, mmHg	Continuous	<0.001	1.006 (0.998-1.013)	0.128
DBP, mmHg	Continuous	0.552	Not significant	
Blood pressure, mmHg	Categorical	0.054	Substituted by continuous	
SBP 120-129 and DBP<80	vs <120 and <80	0.589	Substituted by continuous	
SBP 130-139 or DBP 80-89	vs <120 and <80	0.036	Substituted by continuous	
SBP ≥140 or DBP ≥90	vs <120 and <80	0.013	Substituted by continuous	
Body mass index, kg/m^2	Continuous	0.903	Not significant	
Body mass index	Categorical	0.608	Not significant	
≥23 kg/m^2 and <25 kg/m^2	vs <23 kg/m^2	0.408	Not significant	
≥25 kg/m^2	vs <23 kg/m^2	0.850	Not significant	
Fasting serum glucose, mg/dL	Continuous	0.001	1.004 (1.000-1.007)	0.032
Fasting serum glucose, mg/dL	Categorical	0.001	Substituted by continuous	
≥100 and <126	vs <100	0.069	Substituted by continuous	
≥126	vs <100	<0.001	Substituted by continuous	
Total cholesterol, mg/dL	Continuous	0.048	0.999 (0.996-1.002)	0.388
Total cholesterol, mg/dL	Categorical	0.171	Not significant	
≥200 and <240	vs <200	0.078	Not significant	
≥240	vs <200	0.315	Not significant	
AST, IU/L	Continuous	0.457	Not significant	
ALT, IU/L	Continuous	0.365	Not significant	
γ-GT, IU/L	Continuous	0.002	1.002 (1.000-1.003)	0.016
Charlson comorbidity index	Continuous	<0.001	1.032 (0.992-1.074)	0.123
Smoking status	Categorical	0.479	Not significant	
Previous	vs never	0.505	Not significant	
Current	vs never	0.254	Not significant	
Alcohol consumption	Categorical	0.010	1.000 (Reference)	0.530
1-2 day/week	vs 0 day/week	0.277	1.021 (0.741-1.407)	0.897
3-4 day/week	vs 0 day/week	0.136	1.325 (0.764-2.295)	0.316
≥5 day/week	vs 0 day/week	0.008	1.374 (0.823-2.294)	0.224
Walking	Categorical	0.038	1.000 (Reference)	0.708
1-2 day/week	vs 0 day/week	0.007	0.874 (0.628-1.216)	0.423
3-4 day/week	vs 0 day/week	0.442	1.087 (0.761-1.553)	0.647
≥5 day/week	vs 0 day/week	0.855	0.987 (0.723-1.347)	0.933
MVPA	Categorical	0.002	1.000 (Reference)	0.492
1-2 day/week	vs 0 day/week	<0.001	0.795 (0.573-1.102)	0.169
3-4 day/week	vs 0 day/week	0.046	0.828 (0.587-1.166)	0.279
≥5 day/week	vs 0 day/week	0.338	0.897 (0.559-1.439)	0.651
History of chemotherapy	vs never	0.202	Not significant	
History of radiotherapy	vs never	0.209	Not significant	

HR calculated by Cox proportional hazard regression. Both continuous and categorical levels were analyzed in partial variables to identify significance of linear and non-linear associations. Acronyms: HR, hazard ratio; CI, confidence interval; SBP, systolic blood pressure; DBP, diastolic blood pressure; AST, aspartate

aminotransferase; ALT, alanine aminotransferase; γ-GT, γ-glutamyl transpeptidase; MVPA, moderate-to-vigorous physical activity.

Supplementary Table 5. Univariate and multivariate analysis of factors associated with ischemic stroke

Variable	Input type	Univariate	Multivariate	
		P value	HR (95% CI)	P value
Age, years	Continuous	<0.001	1.081 (1.060-1.103)	<0.001
Sex, female	vs male	0.060	Not significant	
Income, quartile	Categorical	0.672	Not significant	
Second	vs first	0.320	Not significant	
Third	vs first	0.585	Not significant	
Fourth	vs first	0.639	Not significant	
SBP, mmHg	Continuous	<0.001	1.007 (0.997-1.017)	0.191
DBP, mmHg	Continuous	0.466	Not significant	
Blood pressure, mmHg	Categorical	0.019	Substituted by continuous	
SBP 120-129 and DBP<80	vs <120 and <80	0.102	Substituted by continuous	
SBP 130-139 or DBP 80-89	vs <120 and <80	0.006	Substituted by continuous	
SBP ≥140 or DBP ≥90	vs <120 and <80	0.003	Substituted by continuous	
Body mass index, kg/m^2	Continuous	0.562	Not significant	
Body mass index	Categorical	0.116	Not significant	
≥23 kg/m^2 and <25 kg/m^2	vs <23 kg/m^2	0.443	Not significant	
≥25 kg/m^2	vs <23 kg/m^2	0.149	Not significant	
Fasting serum glucose, mg/dL	Continuous	<0.001	1.005 (1.002-1.009)	0.003
Fasting serum glucose, mg/dL	Categorical	0.003	Substituted by continuous	
≥100 and <126	vs <100	0.075	Substituted by continuous	
≥126	vs <100	<0.001	Substituted by continuous	
Total cholesterol, mg/dL	Continuous	0.805	Not significant	
Total cholesterol, mg/dL	Categorical	0.410	Not significant	
≥200 and <240	vs <200	0.283	Not significant	
≥240	vs <200	0.606	Not significant	
AST, IU/L	Continuous	0.226	Not significant	
ALT, IU/L	Continuous	0.556	Not significant	
γ-GT, IU/L	Continuous	0.001	1.002 (1.001-1.004)	0.005
Charlson comorbidity index	Continuous	0.003	1.039 (0.983-1.099)	0.175
Smoking status	Categorical	0.051	1.000 (Reference)	0.010
Previous	vs never	0.094	1.451 (0.998-2.108)	0.051
Current	vs never	0.030	1.946 (1.221-3.100)	0.005
Alcohol consumption	Categorical	0.258	Not significant	
1-2 day/week	vs 0 day/week	0.467	Not significant	
3-4 day/week	vs 0 day/week	0.763	Not significant	
≥5 day/week	vs 0 day/week	0.082	Not significant	
Walking	Categorical	0.152	1.000 (Reference)	0.782
1-2 day/week	vs 0 day/week	0.023	0.818 (0.516-1.299)	0.395
3-4 day/week	vs 0 day/week	0.341	0.947 (0.471-1.570)	0.831
≥5 day/week	vs 0 day/week	0.524	0.834 (0.537-1.294)	0.418
MVPA	Categorical	0.028	1.000 (Reference)	0.681
1-2 day/week	vs 0 day/week	0.003	0.759 (0.471-1.222)	0.256
3-4 day/week	vs 0 day/week	0.249	0.949 (0.593-1.517)	0.826
≥5 day/week	vs 0 day/week	0.634	1.083 (0.567-2.070)	0.810
History of chemotherapy	vs never	0.684	Not significant	
History of radiotherapy	vs never	0.525	Not significant	

HR calculated by Cox proportional hazard regression. Both continuous and categorical levels were analyzed in partial variables to identify significance of linear and non-linear associations. Acronyms: HR, hazard ratio; CI,

confidence interval; SBP, systolic blood pressure; DBP, diastolic blood pressure; AST, aspartate aminotransferase; ALT, alanine aminotransferase; γ-GT, γ-glutamyl transpeptidase; MVPA, moderate-to-vigorous physical activity.

Supplementary Table 6. Univariate and multivariate analysis of factors associated with hemorrhage stroke

Variable	Input type	Univariate	Multivariate	
		P value	HR (95% CI)	P value
Age, years	Continuous	<0.001	1.077 (1.037-1.119)	<0.001
Sex, female	vs male	0.122	Not significant	
Income, quartile	Categorical	0.896	Not significant	
Second	vs first	0.464	Not significant	
Third	vs first	0.783	Not significant	
Fourth	vs first	0.745	Not significant	
SBP, mmHg	Continuous	0.266	Not significant	
DBP, mmHg	Continuous	0.592	Not significant	
Blood pressure, mmHg	Categorical	0.884	Not significant	
SBP 120-129 and DBP<80	vs <120 and <80	0.828	Not significant	
SBP 130-139 or DBP 80-89	vs <120 and <80	0.554	Not significant	
SBP ≥140 or DBP ≥90	vs <120 and <80	0.685	Not significant	
Body mass index, kg/m^2	Continuous	0.354	Not significant	
Body mass index	Categorical	0.399	Not significant	
≥23 kg/m^2 and <25 kg/m^2	vs <23 kg/m^2	0.191	Not significant	
≥25 kg/m^2	vs <23 kg/m^2	0.931	Not significant	
Fasting serum glucose, mg/dL	Continuous	0.794	Not significant	
Fasting serum glucose, mg/dL	Categorical	0.510	Not significant	
≥100 and <126	vs <100	0.246	Not significant	
≥126	vs <100	0.718	Not significant	
Total cholesterol, mg/dL	Continuous	0.349	Not significant	
Total cholesterol, mg/dL	Categorical	0.544	Not significant	
≥200 and <240	vs <200	0.835	Not significant	
≥240	vs <200	0.303	Not significant	
AST, IU/L	Continuous	0.363	Not significant	
ALT, IU/L	Continuous	0.835	Not significant	
γ-GT, IU/L	Continuous	0.315	Not significant	
Charlson comorbidity index	Continuous	0.449	Not significant	
Smoking status	Categorical	0.617	Not significant	
Previous	vs never	0.873	Not significant	
Current	vs never	0.328	Not significant	
Alcohol consumption	Categorical	0.001	1.000 (Reference)	0.002
1-2 day/week	vs 0 day/week	0.401	0.841 (0.319-2.222)	0.727
3-4 day/week	vs 0 day/week	<0.001	5.525 (2.254-13.543)	<0.001
≥5 day/week	vs 0 day/week	0.414	1.603 (0.379-6.788)	0.522
Walking	Categorical	0.644	Not significant	
1-2 day/week	vs 0 day/week	0.966	Not significant	
3-4 day/week	vs 0 day/week	0.354	Not significant	
≥5 day/week	vs 0 day/week	0.348	Not significant	
MVPA	Categorical	0.688	Not significant	
1-2 day/week	vs 0 day/week	0.558	Not significant	
3-4 day/week	vs 0 day/week	0.388	Not significant	
≥5 day/week	vs 0 day/week	0.900	Not significant	
History of chemotherapy	vs never	0.107	Not significant	
History of radiotherapy	vs never	0.576	Not significant	

HR calculated by Cox proportional hazard regression. Both continuous and categorical levels were analyzed in partial variables to identify significance of linear and non-linear associations. Acronyms: HR, hazard ratio; CI,

confidence interval; SBP, systolic blood pressure; DBP, diastolic blood pressure; AST, aspartate aminotransferase; ALT, alanine aminotransferase; γ-GT, γ-glutamyl transpeptidase; MVPA, moderate-to-vigorous physical activity.

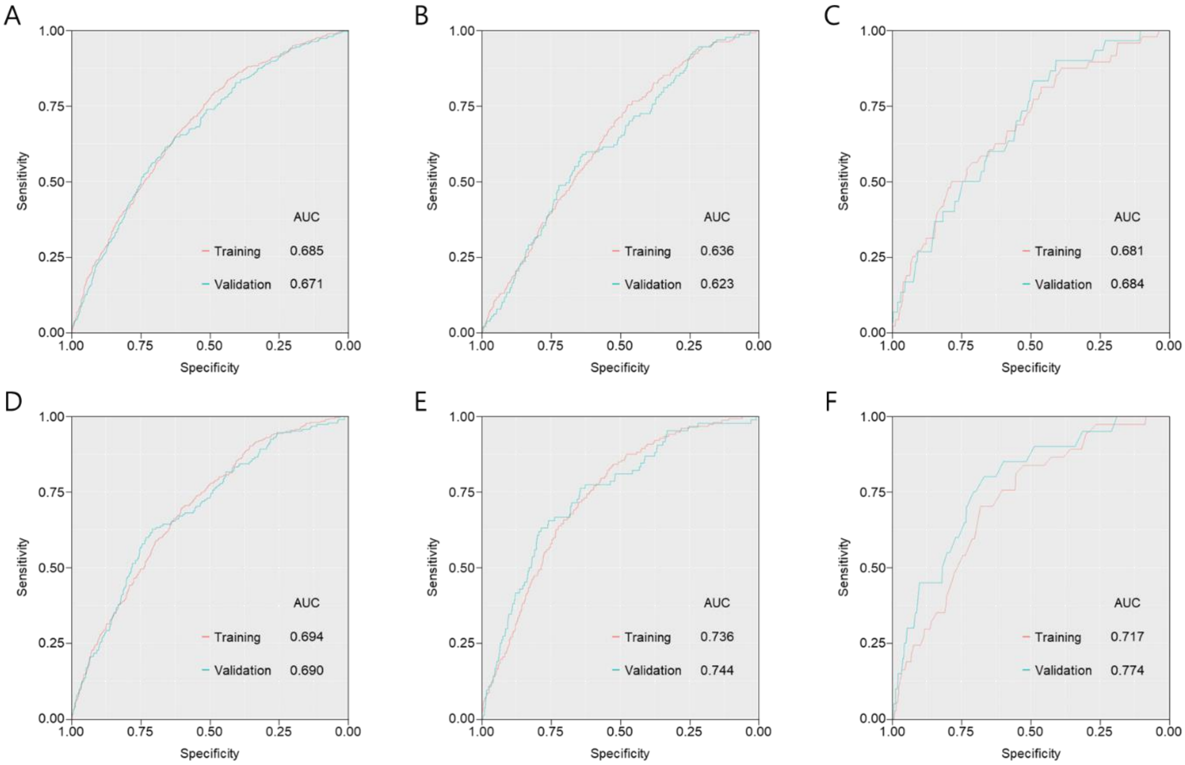

Supplementary Figure 2. Receiver operating curves for training and validation of the predictive nomograms. (A) Overall cardiovascular disease nomogram. (B) Ischemic heart disease nomogram. (C) Myocardial infarction nomogram. (D) Total stroke nomogram. (E) Ischemic stroke nomogram. (F) hemorrhage stroke nomogram.

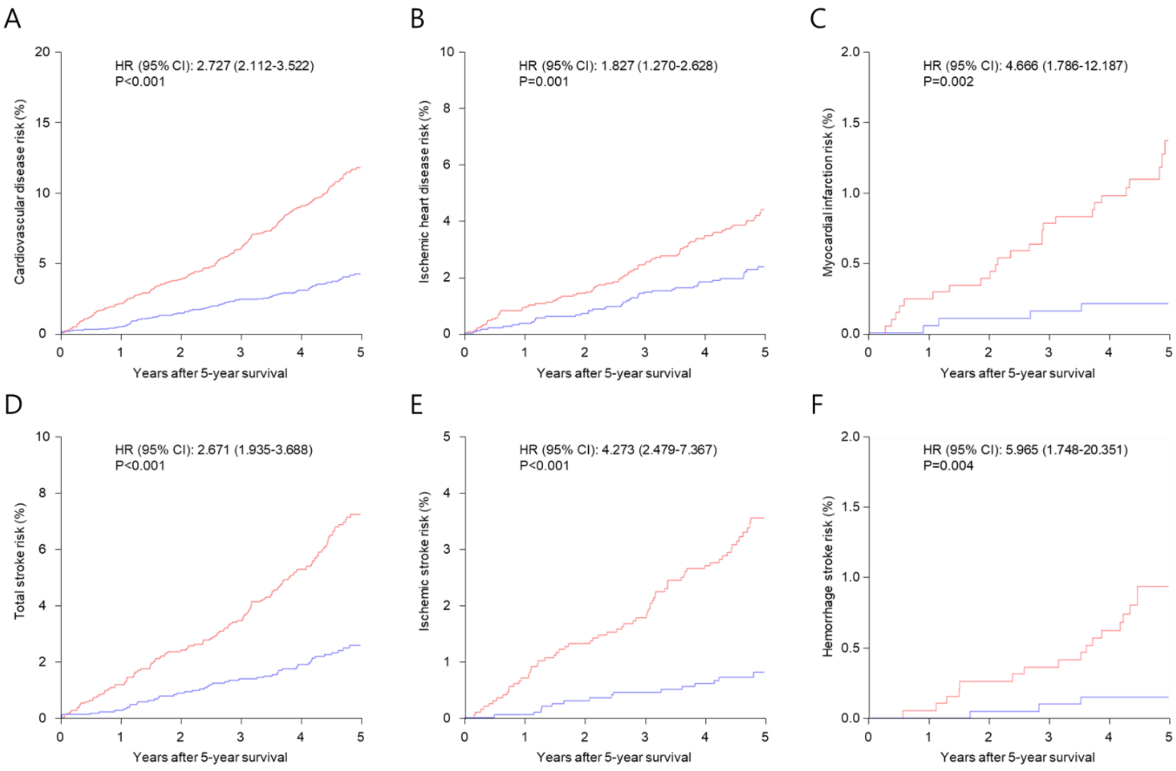

Supplementary Figure 3. Kaplan-Meier estimation of cardiovascular disease risks according to the nomograms-derived dichotomous stratification. High- and low-risk groups stratified according to the median nomogram scores are presented with red and blue curves, respectively. Hazard ratios calculated using the Cox proportional hazards regression. (A) Overall cardiovascular disease risk. (B) Ischemic heart disease risk. (C) Myocardial infarction risk. (D) Total stroke risk. (E) Ischemic stroke risk. (F) hemorrhage stroke risk.
